# Bone Microarchitecture in Obese Postmenopausal Chinese Women: The Chinese Vertebral Osteoporosis Study (ChiVOS)

**DOI:** 10.3389/fendo.2022.891413

**Published:** 2022-07-05

**Authors:** Wenting Qi, Yan Jiang, Wei Liu, Yue Chi, Ruizhi Jiajue, Qianqian Pang, Ou Wang, Mei Li, Xiaoping Xing, Wei Yu, Weibo Xia

**Affiliations:** ^1^ Department of Endocrinology, Key Laboratory of Endocrinology of National Health Commission, State Key Laboratory of Complex Severe and Rare Diseases, Peking Union Medical College Hospital, Chinese Academy of Medical Science and Peking Union Medical College, Beijing, China; ^2^ Department of Radiology, Peking Union Medical College Hospital, Chinese Academy of Medical Science and Peking Union Medical College, Beijing, China

**Keywords:** obesity, BMI - body mass index, postmenopausal women, HR-pQCT (high-resolution peripheral quantitative computed tomography), bone microarchitecture

## Abstract

**Background:**

Obesity is associated with improved bone mass and microarchitecture in Caucasian individuals, but evidence in obese Asian individuals is lacking.

**Objective:**

To analyze the areal bone mineral density (aBMD) and bone microarchitecture in normal-weight, overweight, and obese postmenopausal Chinese women.

**Methods:**

A total of 243 postmenopausal women from the Chinese Vertebral Osteoporosis Study (ChiVOS) were included and were divided into three groups (OB, obese group; OW, overweight group; NW, normal weight group) by BMI level. aBMD, trabecular bone score (TBS), and appendicular lean mass (ALM) were measured by dual‐energy X‐ray absorptiometry (DXA). Bone microarchitecture was measured by HR-pQCT at the distal radius and tibia. X-ray was performed to confirm vertebral fractures (VFs). Multiple linear regression was used to evaluate the correlations between bone parameters and ALM after adjusting for confounding variables.

**Results:**

The prevalence of VFs and clinical fractures were similar among the groups. Participants in the OB group showed a lower level of osteocalcin with comparable levels of other bone turnover markers (BTMs). The aBMD at several skeletal sites was higher in the OB group than in the NW group after adjusting for age (*p*<0.01 for all comparisons). At the radius, the OB group had a higher Ct.Ar, Tb.vBMD, Tb.BV/TV, Tb.N, Tb.Th, and Ct.Th than the NW group after adjusting for covariates (*p*<0.05 for all). Differences of a similar magnitude were found at the distal tibia. There was a trend of decreasing trend in Tb.Sp, Tb.1/N/SD, and Ct.Po among groups at both sites. However, the bone microarchitecture did not differ between participants with severe obesity (BMI≥35.0kg/m^2^) and those with 30.0≤BMI<35 kg/m^2^. Multiple linear regression revealed that the associations between ALM and most of the bone microarchitecture parameters at both sites were much stronger than the association between body weight and bone parameters.

**Conclusion:**

We have observed significant improvements in aBMD, bone geometry, and bone microarchitecture in obese postmenopausal Chinese women. Except for a lower level of osteocalcin in the OB group, no significant differences in BTMs were found among the groups. Compared with body weight, ALM may explain greater variance in the improvement of bone microarchitecture parameters.

## Introduction

Osteoporosis is characterized by lower bone mass and the deterioration of bone microarchitecture with an increased risk of bone fracture, which has already become a worldwide public health problem due to the high morbidity, mortality, and heavy socioeconomic burden. According to the most recent population-based study in China, the prevalence of osteoporosis in men aged 40 years or older was 5.0% (95% CI 4.2%-5.8%), and that in women was 20.6% (95% CI 19.3%-22.0%) (1). For women aged 80 years or older, the prevalence of osteoporosis in China is even higher than that in most American and European countries, with a prevalence of 67.5% (95% CI 56.5%-78.4%) (1–3).

Obesity is an important public health concern worldwide and has a great impact on both mortality and morbidity. Despite being a risk factor for cardiovascular disease and diabetes, the potential protective effect of obesity against osteoporosis is becoming a focus of medical care. Obesity is traditionally considered a protective factor for fractures due to increased loading through bodyweight increasing BMD and the protective effect of soft tissue padding (4). However, the relationship between obesity and the risk of fracture is controversial and appears to vary depending on sex and skeletal sites, especially for vertebral fractures (VFs) (5–7), despite improved areal bone mineral density (aBMD) (8–10).

Since dual‐energy X‐ray absorptiometry (DXA) may overestimate aBMD values at axial sites due to excess fat tissue (11), and other factors including bone geometry and microarchitecture might also be important determinants of bone strength in addition to BMD (12), researchers have performed high-resolution peripheral quantitative computed tomography (HR-pQCT) to measure volumetric densities and the cortical and trabecular compartments in obese individuals, which is less affected by soft tissue padding. Several studies have demonstrated the bone microarchitecture characteristics in obesity, with favorable cortical and trabecular parameters (10, 13, 14). However, data on the bone microarchitecture across the BMI spectrum are lacking in postmenopausal Chinese women. Owing to the different diagnostic criteria and prevalence of obesity from Caucasian individuals, the characteristics of bone microarchitecture in the Chinese population remain unclear.

Regarding this research gap, we conducted a cross-sectional study to evaluate bone microarchitecture in obese postmenopausal Chinese women compared with overweight and normal-weight women from the Chinese Vertebral Osteoporosis Study (ChiVOS). The findings will increase our understanding of obesity and bone health.

## Methods

### Study Design and Participants

We conducted a subgroup study within a nationwide, observational, population-based study to investigate the prevalence of VFs among postmenopausal women in China (ChiVOS). Briefly, the ChiVOS randomly recruited 2,664 community-dwelling postmenopausal women aged 50 or over from 5 regions throughout China. All participants recruited in Beijing (n=274) were enrolled at Peking Union Medical College Hospital (PUMCH, Beijing, China), and underwent an additional HR-pQCT examination. The weight and height of each participant were measured by standard methods, and body mass index (BMI) was calculated as the weight in kilograms divided by height in meters squared. Since low-weight conditions have typically been regarded as an increased risk for fractures (15), we excluded participants with BMI<18.5kg/m^2^ (n=6). After excluding 25 participants who lacked HR-pQCT measurements, 243 participants were finally included in this study. According to the cutoff points of the Chinese criteria (16), women with BMI≥28.0kg/m^2^ were grouped into the obese group (OB, n=73), women with BMI ranging from 24.0 to 27.9 kg/m^2^ were grouped into the overweight group (OW, n=98), and women who had normal BMI, corresponding to a BMI of 18.5-23.9 kg/m^2^, were grouped into the normal-weight group (NW, n=72). Among the obese participants, we further categorized those with BMI≥35kg/m^2^ into the severely obese group (n=9). We also performed a subgroup analysis to investigate whether abdominal obesity (AO) would affect bone microarchitecture, defined as waist circumference (WC) ≥85cm, according to the Health Industry Standards of the People’s Republic of China classifications (17). Participants with a history of anti-osteoporosis drug use (n=5) were not excluded due to the small sample size. This study was approved by the ethics committee of PUMCH, and all participants provided written informed consent prior to engagement in any study activities.

### Clinical Data Collection

Each participant completed an investigator-administered questionnaire, which contained questions regarding demographic information, personal habits and living environment, physical condition, bone status and fracture history, medications, and bone health knowledge. Alcohol intake (never/former/current drinking and average unit/week) and smoking history (never/former/current smoking and average cigarettes/day) were also obtained. Current smoking was defined as smoking at least 1 cigarette/day for more than 6 months, and participants who had quit smoking for more than 6 months when filling out the questionnaire were classified as former smokers. Current drinking was defined as 1 unit of alcohol per week for more than 6 months, and participants who had stopped drinking for more than 6 months when filling out the questionnaire were classified as former drinkers. Information about nutrition and dietary intake included weekly calcium, vitamin D, and milk supplementation status. Information about physical activity (hours and intensity/day) was also collected. A detailed history of self-reported fractures occurring after age 50 was collected. Prior and concomitant medications, history of corticosteroid use, hormone replacement therapy (HRT), and any antiresorptive drugs or teriparatide were recorded (medication name, daily dose, and treatment duration).

### Biochemical Measurements

The samples were collected from each participant in the morning (7-9 am) after fasting for at least 8 hours. The blood samples were centrifuged at 3,000 r/min for 4 minutes to separate the serum for analysis. Measurements of serum calcium (Ca), phosphorus (P), and alkaline phosphatase (ALP) were performed in each participant with a Beckman Automatic Biochemical Analyzer (AU5800, Beckman Coulter, Indianapolis, IN, USA). Bone turnover markers (BTMs) including beta-C-terminal telopeptide (β-CTX), N-terminal propeptide of type I procollagen (P1NP) and osteocalcin were measured by electrochemiluminescence immunoassay (Roche Cobas e601, Mannheim, Germany). Serum total 25‐hydroxyvitamin D (total 25OHD) and parathyroid hormone (PTH) were measured by electrochemiluminescence immunoassay (Roche Cobas e601, Mannheim, Germany) and chemiluminescence (Siemens ADVIA Centaur, Munich, Germany) respectively at the end of enrollment using stored serum samples. The intra-assay coefficients of variability of the main measurements are 2.2% - 2.6% for PTH, 1.1% - 3.1% for 25OHD, 2.7% - 3.1% for β-CTX, and 0.9% - 1.1% for P1NP; The inter-assay coefficients of variability of the main measurements are 2.8% - 5.8% for PTH, 2.2% - 4.3% for 25OHD, 2.9% - 3.4% for β-CTX, and 1.4% - 1.6% for P1NP. All these measurements were made in the central laboratory of PUMCH.

### aBMD, TBS, and Appendicular Lean Mass Measurements

Certified technicians performed DXA on each participant to measure aBMD at the lumbar spine (LS, L1-L4), femur neck (FN), and total hip (TH), using GE-Lunar scanners (GE Healthcare, Madison, WI, USA). Absolute aBMD values were recorded. The trabecular bone score (TBS) was obtained retrospectively using DXA scans at the same region of interest (L1-L4) and TBS iNsight v2.1 software (Medimaps). Quality control and calibration of DXA machines were performed per routine practice at each site. DXA results were segmented into the trunk and limbs automatically by the software to determine the appendicular lean mass (ALM). The skeletal muscle index (SMI) was calculated as the ALM divided by the square of height (m^2^) to indicate body-size normalized data and minimize the correlation between lean mass and height. The coefficient of variance (CV%) of DXA was approximately 0.9% to 1.5% for LS, 0.7% to 1.2% for TH, and 1.6% to 2.1% for FN respectively.

### Ascertainment of VFs

Morphometric VFs were assessed by the lateral radiographs of the thoracolumbar spine in all women at the visit to our center. Two experienced radiologists independently evaluated the radiographs to diagnose VFs according to the semiquantitative (SQ) technique of Genant (18) in a blinded fashion. Radiographic VFs with SQ≥1 were categorized as clinical VFs. Discrepancies in the diagnosis between the radiologists were resolved by reassessment and ultimate consensus.

### Bone Microarchitectural Measurements

All subjects underwent HR-pQCT of the nondominant distal radius and tibia (Xtreme CT II; Scanco Medical AG, Bassersdorf, Switzerland) to measure bone microarchitecture using a standard protocol (19). The isotropic resolution of the images was 61 μm. The reference line was placed at the distal endplate of the nondominant radius and tibia. The first slice of the region of interest (ROI) was 9.0 mm and 22.0 mm proximal to the reference lines of the radius and tibia respectively, with a total of 168 parallel CT slices scanned for the 3D image reconstruction of the nondominant radius and tibia. The parameters consisted of bone geometry indices (Tt.Ar, Tb.Ar and Ct.Ar; Tb.Th and Ct.Th), indices of volumetric BMD (Tt.vBMD, Ct.vBMD and Tb.vBMD), trabecular bone parameters (Tb.BV/TV, Tb.Sp and Tb.N), and cortical porosity (Ct.Po). The CV% of HR-pQCT was 0.7% to 1.5% for Tt.vBMD and Tb.vBMD, 2.5% to 4.4% for trabecular architecture, and 0.9% to 1.5% for the Tt.Ar, Ct.Ar, Ct.Po, Ct.Th, and Ct.vBMD.

### Statistical Analysis

Variables were tested for normality of distribution using the Shapiro-Wilk test. Continuous variables with normal distributions are presented as the means ± SDs, categorical variables are expressed as percentages, and continuous nonnormally distributed data are expressed as medians (interquartile range). Bone parameters were compared among groups by ANOVA and the Kruskal-Wallis H-test for normally and nonnormally distributed data respectively, followed by pairwise comparisons if the p-value for the overall was <0.05. Bonferroni *post hoc* analysis was used for multiple comparisons among groups. Chi-square or Fisher’s exact test was performed for categorical variables. Multiple linear regression analysis was performed to evaluate the correlations between the bone parameters and weight or ALM with adjustment for confounding variables. For subgroup analyses of abdominal obesity (AO), Student’s *t* tests or the chi-square tests were performed to compare parameters between groups. All statistical analyses were performed using IBM SPSS 26.0 software (IBM Corp., Armonk, NY, USA). A p-value<0.05 in a two-tailed test was considered statistically significant for all comparisons.

## Results

### Clinical Characteristics and the Prevalence of VFs

A total of 243 postmenopausal women were recruited for this study. **Table 1** showed the clinical characteristics of the three groups. The women in the OB group were younger than those in the OW group, with medians of 64.0 and 69.0 years old respectively (*p*=0.007). The groups did not differ in terms of height or age of menopause, but the OB and OW groups had markedly elevated height loss compared with NW group (*p*=0.047 and *p*=0.015, respectively). Obviously, weight, waistline, and BMI were highest in the OB group and lowest in the NW group, with the highest BMI being 37.2 kg/m^2^. Women in the OB group also showed higher ALM and SMI than those in the other two groups (*p*<0.001 for all comparisons). No significant differences were noted among groups in terms of fall risk regardless of whether the falls occurred in the last year or after age 50. Regarding fracture prevalence, the proportion of individuals with VFs was similar among the groups (16.44% vs. 18.37% vs. 13.89% in the OB, OW and NW groups respectively). Previous fractures (traumatic or nontraumatic) were reported in 5.48% of the OB group vs. 3.06% of the OW and 4.17% of the NW group, with no significant difference among groups. Smoking habits, alcohol intake, physical activity, and calcium, vitamin D, and milk supplementation status were comparable among groups. There was no significant difference in medication use among the groups.

**Table 1:** Clinical characteristics in normal-weight (NW), overweight (OW), and obese (OB) postmenopausal women.

	NW (n=72)	OW (n=98)	OB (n=73)	ANOVA *p*-value	*p1*	*p2*	*p3*
Age (years)	66.5 (59.0-74.0)	69.0 (64.0-76.25)	64.0 (59.0-69.0)	**0.007**	0.164	0.881	**0.007**
Height (cm)	156.28 ± 6.51	154.42 ± 5.63	154.8 (149.5-159.2)	0.133	/	/	/
Weight (kg)	53.82 ± 5.54	62.02 ± 5.37	74.65 ± 9.15	**<0.0001**	**<0.0001**	**<0.0001**	**<0.0001**
Waistline (cm)	80.02 ± 6.67	89.8 (85.75-94.1)	99.31 ± 8.83	**<0.0001**	**<0.0001**	**<0.0001**	**<0.0001**
BMI (kg/m^2^)	22.45 (21.1-23.3)	26.05 (24.90-26.73)	30.6 (28.65-32.75)	**<0.0001**	**<0.0001**	**<0.0001**	**<0.0001**
Age of menopause (years)	49.95 ± 3.44	50.0 (47.0-53.0)	50.0 (48.0-53.0)	0.765	/	/	/
Height loss (cm)	2.6 (1.8-4.8)	3.7 (2.65-5.65)	4.20 ± 2.61	**0.011**	**0.015**	**0.047**	>0.99
Falls (happened after age 50), n (%)	31/72 (43.1%)	40/98 (40.8%)	31/71 (43.7%)	/	>0.99	>0.99	>0.99
Falls (happened in the last year), n (%)	14/72 (19.4%)	17/98 (17.3%)	15/73 (20.5%)	/	>0.99	>0.99	>0.99
Any kinds of fractures (≥2 happened after age 50), n (%)	3/72 (4.17%)	3/98 (3.06%)	4/73 (5.48%)	/	0.699	>0.99	>0.99
Vertebral fractures, n (%)	10/72 (13.89%)	18/98 (18.37%)	12/73 (16.44%)	/	0.437	0.669	0.743
Smoking habit				/			
Former smoking, n (%)	2/72 (2.78%)	5/98 (5.10%)	1/73 (1.37%)	/	0.70	0.62	0.241
Current smoking, n (%)	1/72 (1.39%)	1/98 (1.02%)	2/73 (2.74%)	/	0.575	>0.99	0.575
Alcohol intake of more than 1 unit/week, n (%)	1/72 (1.39%)	6/98 (6.12%)	2/73 (2.74%)	/	0.241	>0.99	0.469
Calcium supplements, n (%)	24/72 (33.33%)	28/98 (28.57%)	22/73 (30.14%)	/	0.614	0.723	0.866
Vitamin D supplements, n (%)	18/72 (25.0%)	17/98 (17.35%)	12/73 (16.44%)	/	0.252	0.224	>0.99
Milk drinking 3 and more times/week, n (%)	54/72 (75.0%)	72/98 (73.47%)	44/73 (60.27%)	/	0.861	0.076	0.097
More than one hour of moderate intensity physical activity/day, n (%)	48/72 (66.67%)	56/98 (57.14%)	37/73 (50.68%)	/	0.265	0.064	0.44
Long time use of glucocorticoid (≥3 months), n (%)	4/71 (5.63%)	2/96 (2.08%)	5/73 (6.85%)	/	0.403	>0.99	0.241
Long time use of HRT (≥3 months), n (%)	5/71 (7.04%)	5/98 (5.10%)	3/73 (4.11%)	/	0.744	0.491	>0.99
Anti-osteoporosis drugs (≥3 months), n (%)	3/72 (4.17%)	1/98 (1.02%)	3/73 (4.11%)	/	0.312	>0.99	0.314
ALM (kg)	14.10 ± 1.56	15.0 ± 1.51	17.18 (15.58-18.42)	**<0.0001**	**0.01**	**<0.0001**	**<0.0001**
SMI (kg/m^2^)	5.77 ± 0.48	6.29 ± 0.43	7.13 ± 0.71	**<0.0001**	**<0.0001**	**<0.0001**	**<0.0001**

1Data presented as mean±SD or median (interquartile range) or percentages.

2Significant values are shown in bold. p1: NW vs. OW, p2: NW vs. OB, p3: OW vs. OB.

3BMI, body mass index; HRT, hormone-replacement therapy; ALM, appendicular lean mass; SMI, skeletal muscle index [SMI = ALM (kg) / height^2^ (m^2^)].

### Comparison of aBMD, TBS, and BTMs

**Table 2** showed aBMD, TBS, and BTM measurements for all participants. When stratifying the population by BMI status, the aBMD at several skeletal sites showed a trend of being highest in the OB group, followed by the OW group, and lowest in the NW group, but there was no significant difference between the OW and NW groups. After adjusting for age, differences in aBMD between the OB and NW groups persisted. In contrast, the TBS values were comparable among the groups. The median (interquartile range) values in the OB group for total 25OHD were lower than those in the NW group (13.62[11.08-17.60] vs. 16.75[14.6-23.4] ng/ml, *p*=0.001), and the OB group had statistically higher PTH levels (43.85[31.52-57.83] vs. 35.53 ± 10.26 pg/ml, *p=*0.001). For other BTMs, a lower level of osteocalcin was found in the OB group than in the OW and NW groups (*p*<0.05 for all), but no significant differences in serum levels of ALP, β-CTX or P1NP were found among the groups.

**Table 2:** Age-adjusted measurements of aBMD and biochemical characteristics in normal-weight (NW), overweight (OW), and obese (OB) postmenopausal women.

	NW(n=72)	OW(n=98)	OB (n=73)	ANOVA *p*-value	*p1*	*p2*	*p3*
Lumbar spine aBMD (g/cm^2^)	0.98 (0.86-1.09)	1.03 (0.93-1.15)	1.14 ± 0.18	**<0.0001**	0.078	<0.0001*	0.005*
Femur neck aBMD (g/cm^2^)	0.76 ± 0.11	0.76 ± 0.12	0.83 ± 0.14	**0.003**	>0.99	0.017*	0.005
Total hip aBMD (g/cm^2^)	0.82 ± 0.12	0.82 (0.72-0.91)	0.92 ± 0.16	**<0.0001**	>0.99	<0.0001*	<0.0001
TBS (L1-L4)	1.30 ± 0.07	1.30 ± 0.087	1.31 ± 0.105	0.92	/	/	/
Ca (mmol/L)	2.32 ± 0.06	2.32 ± 0.08	2.31 ± 0.08	0.643	/	/	/
P (mmol/L)	1.24 ± 0.11	1.20 ± 0.13	1.16 ± 0.15	**0.018**	0.664	**0.014**	0.215
ALP (U/L)	79.0 (66.25-86.75)	82.0 (71.0-98.25)	83.0 (72.0-99.0)	0.330	/	/	/
Total 25OHD (ng/ml)	16.75 (14.6-23.4)	15.1 (12.26-19.15)	13.62 (11.08-17.60)	**0.001**	**0.034**	**0.001**	0.419
PTH (pg/ml)	35.53 ± 10.26	38.11 (30.50-46.16)	43.85 (31.52-57.83)	**0.001**	0.163	**0.001**	0.124
β-CTX (ng/ml)	0.36 (0.24-0.44)	0.38 (0.25-0.46)	0.34 ± 0.14	0.393	/	/	/
P1NP (ng/ml)	53.14 (41.52-69.56)	54.8 (40.70-66.35)	51.83 ± 18.50	0.433	/	/	/
Osteocalcin (ng/ml)	16.83 (12.97-21.16)	16.54 (12.42-20.45)	14.56 (11.22-17.77)	**0.013**	>0.99	**0.025**	**0.036**

1Data presented as mean±SD or median (interquartile range) or percentages.

2Significant values are shown in bold. p1: NW vs. OB, p2: NW vs. OB, p3: OW vs OB. *p<0.05 adjusted for age, current smoking, alcohol intake, supplements of calcium or vitamin D, milk drinking, and physical activity.

3aBMD, areal bone mineral density; TBS, trabecular bone score; ALP, alkaline phosphatase; total 25OHD, total 25-hydroxyvitamin D; PTH, parathyroid hormone; β-CTX, beta-C-terminal telopeptide; P1NP, N-terminal propeptide of type I procollagen.

4Normal reference ranges: Ca: 2.13-2.70mmol/L; P: 0.81-1.45mmol/L; ALP: 50–135U/L; total 25OHD: 30-50ng/ml; PTH: 15.0-65.0pg/ml; β-CTX: menopause to 70y 0.10–0.79ng/ml, >70y 0.11–0.86ng/ml; P1NP: for premenopausal women 15.1-58.6ng/ml.

### Bone Microarchitecture in Normal-Weight, Overweight, and Obese Women

**Figure 1:**
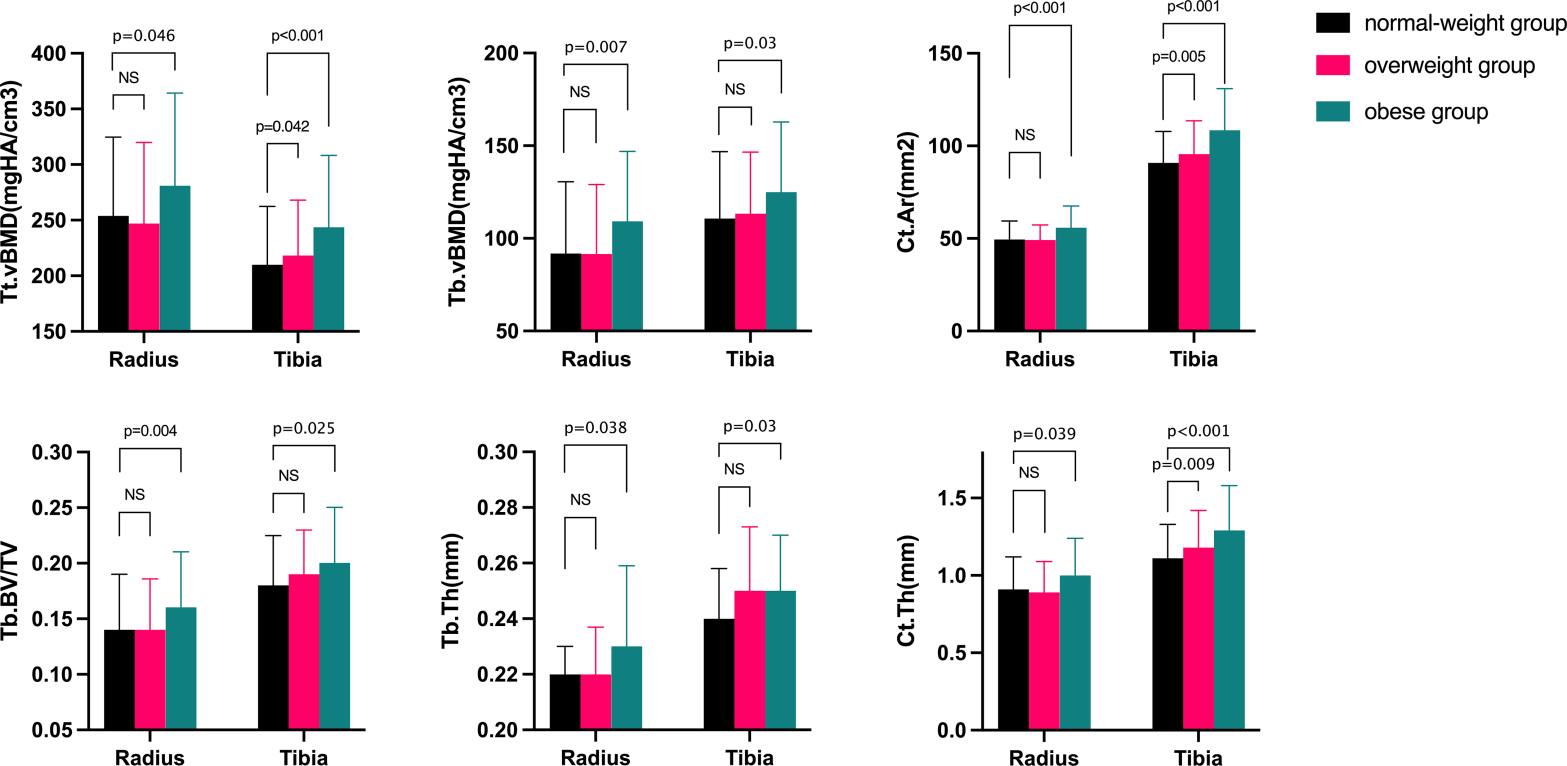
Comparisons of bone microarchitecture at the distal radius and tibia after adjusting for confound variables in normal-weight, overweight, and obese women. Ct.Ar=cortical area; Tt.vBMD=total volume bone mineral density; Tb.vBMD=trabecular volume bone mineral density; Tb.BV/TV=trabecular bone volume fraction; Tb.N=trabecular number; Tb.Th=trabecular thickness; Ct.Th=cortical thickness. The p-value was adjusted for age, current smoking, alcohol intake, supplementations of calcium or vitamin D, milk drinking, and physical activity. NS, no significant.

**Table 3** showed the characteristics of geometric features, vBMD, and microarchitecture measurements at the distal radius and tibia obtained by HR-pQCT. After adjusting for confounding variables, the OB group showed favorable parameters in both the cortical and trabecular bone. At the distal radius, OB women had 14.79% higher Tt.vBMD and 24.06% higher Tb.vBMD than NW women, which were less improved in OW women. No statistically significant difference was found in Ct.vBMD among the groups. Both Tt.Ar and Ct.Ar were highest in the OB groups, followed by the OW group, and they were lowest in NW women. Notably, Ct.Ar was 14.95% higher in the OB group than in the NW group (*p<*0.001). The trabecular bone parameters Tb.BV/TV and Tb.N increased progressively from NW to OW to OB (*p* < 0.05 for all), and Tb.Sp tended to decrease from NW to OB. The same trend was observed for Tb.1/N.SD from OB to NW. For cortical bone, Ct.Th was greater in the OB group than in the OW group (*p* = 0.039). Similar results were found at the distal tibia for Ct.Ar, Tb.vBMD, Tb.BV/TV, Tb.Th, and Ct.Th, which were notably increased in the OB group (*p*<0.05 for all comparisons). Tt.vBMD was also significantly increased as BMI category increased. Tb.N, Tb.Sp, and Tb.1/N.SD were comparable among groups at the distal tibia. No significant differences were detected in Tb.Ar and Ct.Po among the groups at either sites (**Figure 1**).

**Table 3:** Adjusted comparisons of microarchitecture characteristics in normal-weight (NW), overweight (OW), and obese (OB) postmenopausal women at the radius and tibia.

	NW (n=72)	Radius	*#p*	OB (n=73)	*##p*	NW (n=72)	Tibia	*#p*	OB (n=73)	*##p*
		OW (n=98)					OW (n=98)			
Tt.Ar (mm^2^)	239.98 ± 31.39	250.15 ± 42.78	0.197	255.20 ± 44.52	**0.015**	650.22 ± 91.69	647.41 ± 92.81	0.792	669.23 ± 110.20	0.242
Tb.Ar (mm^2^)	194.62 ± 33.05	203.78 ± 44.76	0.355	202.45 ± 45.70	0.147	564.99 ± 94.83	559.04 ± 99.12	0.512	562.47 ± 114.56	0.939
Ct.Ar (mm^2^)	48.5 (42.1-54.0)	49.14 ± 8.14	0.50	55.75 ± 11.87	**<0.001**	88.65 (80.05-99.25)	95.59 ± 17.91	**0.005**	108.38 ± 22.61	**<0.001**
Tt.vBMD (mg HA/cm^3^)	244.7 (203.9-295.9)	247.02 ± 72.84	0.713	280.90 ± 83.28	**0.046**	209.89 ± 52.54	218.13 ± 49.93	**0.042**	243.71 ± 64.42	**<0.001**
Tb.vBMD (mg HA/cm^3^)	84.8 (66.6-110.15)	91.50 ± 37.62	0.477	105.20 (82.8-130.2)	**0.007**	110.63 ± 36.18	113.34 ± 33.26	0.327	124.98 ± 37.80	**0.03**
Ct.vBMD (mg HA/cm^3^)	888.34 ± 68.85	881.4 (833.45-915.55)	0.399	908.3 (825.3-956.4)	0.941	825.59 ± 59.47	818.08 ± 69.71	0.745	847.58 ± 75.63	0.057
Tb.BV/TV	0.13 (0.11-0.16)	0.13 (0.11-0.17)	0.365	0.15 (0.13-0.19)	**0.004**	0.18 (0.15-0.22)	0.19 ± 0.04	0.313	0.20 ± 0.05	**0.025**
Tb.N (1/mm)	1.06 ± 0.27	1.07 ± 0.31	0.341	1.23 (1.02-1.38)	**0.007**	1.08 ± 0.22	1.07 (0.97-1.26)	0.397	1.15 (1.03-1.28)	0.227
Tb.Th (mm)	0.22 ± 0.01	0.22 (0.21-0.23)	0.777	0.22 (0.21-0.23)	**0.038**	0.24 (0.23-0.25)	0.24 (0.23-0.26)	0.137	0.25 ± 0.02	**0.03**
Tb.Sp (mm)	0.91 (0.77-1.13)	0.89 (0.79-1.19)	0.756	0.80 (0.71-0.97)	0.288	0.9 (0.8-1.09)	0.93 (0.78-1.03)	0.961	0.86 (0.77-0.96)	0.648
Tb.1/N.SD (mm)	0.38 (0.29-0.57)	0.36 (0.31-0.58)	0.909	0.34 (0.27-0.41)	0.629	0.38 (0.32-0.48)	0.38 (0.31-0.46)	0.755	0.36 (0.32-0.43)	0.592
Ct.Th (mm)	0.91 (0.76-1.0)	0.89 ± 0.20	0.916	1.04 (0.77-1.17)	**0.039**	1.11 ± 0.22	1.18 ± 0.24	**0.009**	1.29 ± 0.29	**<0.001**
Ct.Po (%)	0.7 (0.4-1.0)	0.8 (0.4-1.1)	0.61	0.60 (0.30-0.80)	0.134	3.4 (2.5-4.5)	3.55 (2.70-4.73)	0.749	3.0 (2.1-4.2)	0.19

1Data presented as mean±SD or median (interquartile range).

2Significant values are shown in bold. #p comparison between the normal-weight group and over-weight group after adjusting age, current smoking, alcohol intake, supplements of calcium or vitamin D, milk drinking, and physical activity; ##p comparison between the normal-weight group and obese group after adjusting age, current smoking, alcohol intake, supplements of calcium or vitamin D, milk drinking, and physical activity.

3Tt.Ar, total area; Ct.Ar, cortical area; Tb.Ar, trabecular area; Tt.vBMD, total volume bone mineral density; Tb.vBMD, trabecular volume bone mineral density; Ct.vBMD, cortical volume bone mineral density; Tb.BV/TV, trabecular bone volume fraction; Tb.N, trabecular number; Tb.Th, trabecular thickness; Tb.Sp, trabecular separation; Tb.1/N.SD, trabecular inhomogeneity of network; Ct.Th, cortical thickness; Ct.Po, cortical porosity.

To evaluate the effect of severe obesity on bone microarchitecture, we further compared the HR-pQCT parameters of severely obese women (n=9), with those of age- and height-matched moderately obese women (n=9), who had BMIs from 30.0 to 35 kg/m^2^. **Table 4** shows the comparisons between the groups. The two groups had similar ages (69.67 ± 10.34 vs. 69.56 ± 9.68, p=0.982) and comparable heights (154.92 ± 6.74 vs. 154.81 ± 3.46, p=0.965). When compared with the age- and height-matched controls, the bone microstructure parameters were not further improved as BMI gradually tends to severe obesity (defined as BMI≥35.0kg/m^2^). Severely obese women shared comparable values of Ct.Ar, Tb.vBMD, Tb.BV/TV, Tb.Th, and Ct.Th with moderately obese women.

**Table 4:** Comparisons of bone microarchitecture in severely obese women and age- and height-matched moderately obese women.

	BMI≥35 (n=9)	Radius	*p*	BMI≥35 (n=9)	Tibia	*p*
		30≤BMI<35 (n=9)			30≤BMI<35 (n=9)	
Tt.Ar (mm^2^)	256.57 ± 42.50	259.03 ± 35.06	0.895	668.92 ± 137.56	689.6 ± 70.41	0.693
Tb.Ar (mm^2^)	204.37 ± 43.89	205.98 ± 36.71	0.934	567.12 ± 133.57	580.16 ± 81.46	0.806
Ct.Ar (mm^2^)	55.80 ± 12.59	56.72 ± 11.13	0.871	107.02 ± 18.06	114.77 ± 21.28	0.417
Tt.vBMD (mg HA/cm^3^)	280.64 ± 79.35	289.64 ± 75.80	0.809	251.89 ± 57.80	254.23 ± 53.45	0.93
Tb.vBMD (mg HA/cm^3^)	116.99 ± 36.80	122.59 ± 37.44	0.753	142.21 ± 39.85	137.64 ± 34.13	0.797
Ct.vBMD (mg HA/cm^3^)	872.28 ± 82.32	885.59 ± 111.52	0.777	830.93 ± 53.47	834.58 ± 74.50	0.907
Tb.BV/TV	0.17 ± 0.052	0.18 ± 0.056	0.722	0.23 ± 0.051	0.22 ± 0.044	0.826
Tb.N (1/mm)	1.30 ± 0.24	1.32 ± 0.16	0.868	1.27 ± 0.20	1.22 ± 0.20	0.597
Tb.Th (mm)	0.22 ± 0.011	0.22 ± 0.016	0.895	0.25 ± 0.012	0.25 ± 0.015	0.62
Tb.Sp (mm)	0.79 ± 0.16	0.75 ± 0.11	0.572	0.80 ± 0.13	0.83 ± 0.15	0.578
Tb.1/N.SD (mm)	0.34 ± 0.082	0.30 ± 0.048	0.21	0.34 ± 0.060	0.36 ± 0.065	0.588
Ct.Th (mm)	0.98 ± 0.25	1.00 ± 0.23	0.817	1.28 ± 0.20	1.35 ± 0.26	0.573
Ct.Po (%)	0.83 ± 0.58	0.72 ± 0.25	0.606	0.038 ± 0.013	3.57 ± 0.75	0.662

1Data presented as mean±SD or median (interquartile range).

2Significant values are shown in bold.

3Tt.Ar, total area; Ct.Ar, cortical area; Tb.Ar, trabecular area; Tt.vBMD, total volume bone mineral density; Tb.vBMD, trabecular volume bone mineral density; Ct.vBMD, cortical volume bone mineral density; Tb.BV/TV, trabecular bone volume fraction; Tb.N, trabecular number; Tb.Th, trabecular thickness; Tb.Sp= trabecular separation; Tb.1/N.SD, trabecular inhomogeneity of network; Ct.Th, cortical thickness; Ct.Po, cortical porosity.

### Associations of HR-pQCT Parameters With Body Weight and ALM

To evaluate the role of lean mass in the increase in absolute values of bone microarchitecture parameters, we investigated the relationship between HR-pQCT parameters and ALM or body weight in postmenopausal women as shown in **Table 5**. There were universal associations between most of the bone microarchitecture parameters and body weight after adjusting for confounding variables. Bodyweight was positively associated with bone geometry indices, Tb.vBMD, trabecular bone parameters Tb.BV/TV, Tb.N, and cortical thickness at both sites (*p*<0.05 for all comparisons), and negatively correlated with Tb.Sp and Tb.1/N.SD at the radius and tibia (*p*<0.01 for all). However, no significant association was detected between body weight and Ct.vBMD or Ct.Po. Interestingly, the association was much stronger between HR-pQCT parameters and ALM than that between HR-pQCT parameters and body weight, indicating the much larger impact of ALM on bone microarchitecture. At the distal tibia site, the effect of ALM on bone geometry was even more pronounced than at the non-weight‐bearing radius. Trabecular bone parameters were positively associated with lean mass with the same magnitude at both sites (*p*<0.05 for all comparisons).

**Table 5:** Correlation of bone microarchitecture with weight and ALM in postmenopausal women (n=243).

	Radius				Tibia			
	Weight		ALM		Weight		ALM	
	β (95%CI)	*p*	β (95%CI)	*p*	β (95%CI)	*p*	β (95%CI)	*p*
Tt.Ar (mm^2^)	1.48 (1.02, 1.94)	**<0.001**	8.71 (6.59, 10.83)	**<0.001**	4.12 (3.01, 5.22)	**<0.001**	24.90 (19.98, 29.82)	**<0.001**
Tb.Ar (mm^2^)	1.15 (0.67, 1.64)	**<0.001**	7.13 (4.90, 9.37)	**<0.001**	3.27 (2.08, 4.46)	**<0.001**	21.40 (16.02, 26.78)	**<0.001**
Ct.Ar (mm^2^)	0.31 (0.21, 0.41)	**<0.001**	1.60 (1.11, 2.08)	**<0.001**	0.65 (0.44, 0.86)	**<0.001**	2.83 (1.80, 3.86)	**<0.001**
Tt.vBMD (mg HA/cm^3^)	0.70 (-0.095, 1.50)	0.084	2.88 (-0.96, 6.72)	0.141	0.93 (0.32, 1.54)	**0.003**	2.91 (-0.048, 5.87)	0.054
Tb.vBMD (mg HA/cm^3^)	0.84 (0.42, 1.27)	**<0.001**	4.31 (2.28, 6.35)	**<0.001**	0.92 (0.52, 1.33)	**<0.001**	3.80 (1.84, 5.77)	**<0.001**
Ct.vBMD (mg HA/cm^3^)	-0.10 (-0.94, 0.73)	0.807	-0.17 (-4.17, 3.83)	0.933	0.12 (-0.62, 0.87)	0.747	0.468 (-3.11, 4.05)	0.797
Tb.BV/TV	0.001 (0.0, 0.002)	**<0.001**	0.005 (0.003, 0.008)	**<0.001**	0.001 (0.001, 0.002)	**<0.001**	0.005 (0.002, 0.007)	**<0.001**
Tb.N (1/mm)	0.008 (0.005, 0.011)	**<0.001**	0.04 (0.024, 0.056)	**<0.001**	0.006 (0.004, 0.009)	**<0.001**	0.03 (0.017, 0.043)	**<0.001**
Tb.Th (mm)	0.0 (0.0, 0.0)	0.117	0.001 (-0.001, 0.002)	0.268	0.0 (0.0, 0.0)	0.129	0.001 (-0.001, 0.002)	0.511
Tb.Sp (mm)	-0.009 (-0.014, -0.004)	**<0.001**	-0.047 (-0.071, -0.024)	**<0.001**	-0.007 (-0.011, -0.004)	**<0.001**	-0.036 (-0.053, -0.018)	**<0.001**
Tb.1/N.SD (mm)	-0.007 (-0.011, -0.002)	**0.003**	-0.035 (-0.056, -0.013)	**0.002**	-0.005 (-0.009, -0.001)	**0.012**	-0.032 (-0.051, -0.012)	**0.002**
Ct.Th (mm)	0.003 (0.0, 0.005)	**0.027**	0.012 (0.001, 0.023)	**0.03**	0.004 (0.001, 0.007)	**0.017**	0.009 (-0.005, 0.024)	0.212
Ct.Po (%)	0.0 (0.0, 0.0)	0.328	0.0 (0.0, 0.0)	0.728	0.0 (0.0, 0.0)	0.333	-0.001 (-0.002, 0.0)	**0.046**

1Significant values are shown in bold.

2p-value was adjusted for age, current smoking, alcohol intake, supplements of calcium or vitamin D, milk drinking, and physical activity by multiple linear regression.

3ALM, appendicular lean mass; Tt.Ar, total area; Ct.Ar, cortical area; Tt.vBMD, total volume bone mineral density; Tb.vBMD, trabecular volume bone mineral density; Ct.vBMD, cortical volume bone mineral density; Tb.BV/TV, trabecular bone volume fraction; Tb.N, trabecular number; Tb.Th, trabecular thickness; Tb.Sp, trabecular separation; Tb.1/N.SD, trabecular inhomogeneity of network; Ct.Th, cortical thickness.

### Subgroup Analysis in Abdominal Obesity Classified by Waist Circumference

Since a few studies have suggested a relationship between abdominal obesity and bone health (20, 21), we conducted a subgroup study to examine the effect of abdominal obesity (AO) on bone microarchitecture. The women with AO showed higher aBMD at the lumbar spine and total hip with increased TBS (**Supplemental Table 2**), but no difference in VFs prevalence was found between groups (**Supplemental Table 1**). Similar to OB vs. NW women, women with AO had lower total 25OHD (14.78 [12.0-19.03] versus 16.58 [13.25-23.5] ng/ml, *p*=0.009) and statistically higher levels of PTH (39.21 [31.56-48.34] versus 34.99 [28.47-44.87] pg/ml, *p* = 0.02), and decreased osteocalcin (15.03[11.53-19.58] versus 17.81[14.26-21.51] ng/ml, *p*=0.003) (**Supplemental Table 2**). We also compared the bone microarchitecture parameters of AO and non-AO groups, but only individual parameters showed differences between groups (**Supplemental Table 3**).

## Discussion

In this cross‐sectional study, we found that obese postmenopausal Chinese women had favorable aBMD and vBMD, and higher values of cortical and trabecular parameters at both the radius and tibia than normal-weight postmenopausal women. However, the bone parameters were not further improved as BMI gradually increased to severe obesity. To the best of our knowledge, this is the first analysis of bone microarchitecture in normal-weight, overweight, and obese Asian women.

Obesity has traditionally been regarded as having a beneficial effect on bone density and fracture risks. We also found increased aBMD at several skeletal sites in the OB group, which indicated the adaptations to the excess body weight. Despite the higher aBMD at the LS, postmenopausal women with obesity did not show a decreased prevalence of clinical fractures, suggesting the importance of evaluating the bone microarchitecture in individuals with obesity. Sornay-Rendu et al. (10) reported greater volumetric density, greater trabecular number with lower trabecular separation, and higher Ct.Ar at the distal radius and tibia in obese postmenopausal France women previously. These results were further confirmed by other studies (9, 22). However, the evidence on bone microarchitecture in obese Asian women is insufficient. Our study demonstrated that OB postmenopausal Chinese women had favorable bone microarchitecture parameters compared with NW women, which was in agreement with the results of previous studies in Caucasian individuals (9, 10, 22). Consistent with these results, we also found greater Tb.vBMD as a result of higher Tb.BV/TV and Tb.N values. Evans and colleagues (9) found similar results, reporting a greater amount of trabecular bone and Tb.BV/TV at both the radius and tibia, but similar trabecular thickness, in favor of OB women compared with NW women (55-75 years old). We also noticed a decreasing trend in Tb.Sp at both sites as BMI category increased, as previously described (9), although this finding was not statistically significant. With a significant trend for increasing Ct.Ar and Ct.Th from NW to OB status, our results are generally in agreement with existing literature indicating that OB women had higher Ct.Ar and Ct.Th values. However, we did not find differences in Ct.Po or Ct.vBMD as previously reported (9, 10). Racial differences may partly account for the discrepancy since Chinese women had thicker and denser cortices than white women, and the thicker cortices and more plate-like trabecular bone tended to persist with aging (23). These advantages may help to narrow the bone microarchitecture gap between OB and NW Chinese women. In addition, OB women in our study had a lower BMI than those in previous studies (30.6[28.65-32.75] vs. 33.4 ± 3.5, 35.9 ± 5.0, 44.8 ± 0.9 kg/m^2^, respectively) (9, 10, 22), owing to the different prevalence of obesity between Chinese and Caucasian individuals. The positive association between body weight and Tb.vBMD, Tb.N, Ct.Th and negative association between body weight and Tb.Sp further confirmed the improved bone mass in OB women to be a result of the adaptive effect of increased body weight, which was similar to results in several other studies (13, 22, 24).

Although obesity is believed to be beneficial to bone health, recent studies have shown that there might be a suboptimal adaptation of BMD to greater body weight (9, 22), and morbid obesity could even reverse the improvement of some tibia parameters (14). These studies provide insights into the complex relationship between obesity and bone health. Data from the UK arm of the GLOW cohort demonstrated that the improved bone microarchitecture was reversed in morbid obesity (BMI≥35kg/m^2^ with hypertension or diabetes) with a decrease in several bone parameters especially the trabecular compartment Tb.vBMD, indicating the limited protection of obesity on bone strength (14). We selected participants with BMI≥35kg/m^2^ as the severely obese group, and the bone parameters were not further improved as BMI gradually increased to severe obesity. Similarly, Dytfeld and colleagues (25) demonstrated that there was a parabolic relationship between BMI and aBMD in postmenopausal women. LS-BMD was highest in OW (BMI from 25 to 30 kg/m^2^) women compared with NW or OB women. The GLOW cohort indicated that increased pelvic fracture risk could be seen at both extremes of BMI within a nonlinear model. The log-hazard for pelvic fracture dropped rapidly from the minimum BMI value to the minimum log-hazard at approximately BMI=30 kg/m^2^ and then rose gradually (26). Owing to the lack of enough participants with BMI over 35 kg/m^2^, expanded samples are required in future studies to investigate the possibility that bone parameters would reach a plateau and even drop gradually at a certain BMI value.

The effect of body weight on bone strength is attributable to both lean mass and fat mass. The positive associations between fat mass and aBMD (27) or bone microarchitecture parameters at the weight‐bearing tibia (10) indicated the direct mechanical effect of fat on bone. However, the positive correlation between fat mass and bone mass was reversed when removing the mechanical loading effect of body weight as previously reported (28), suggesting a detrimental effect of fat mass on bone. Conversely, the effect of lean mass on bone strength was much more pronounced than that of fat mass. In a longitudinal study, the absolute value of bone strength at the tibia seemed to be higher in OW children, but bone strength was adapted to the greater muscle instead of the excess of body fat (29). Sukumar and colleagues (24) revealed that lean mass explained greater variance than fat mass in geometric and strength indices of the distal tibia. Consistent with previous studies, we observed a universal positive association between bone parameters and appendicular lean mass not only at the tibia but also at the radius, which was much stronger than those with body weight. Altogether, these findings suggested that the positive effect of weight on bone appears to be mainly due to the relatively increased muscle mass. However, because obesity is associated with a lower increase in lean mass compared with fat mass, the improvements in bone strength are not adapted to the excess fat mass. The relatively lower level of lean mass in OB participants could partly explain why the bone strength changes in OB women are inappropriate to the excess body weight.

Although conflicting data have been reported, there is considerable evidence supporting that BMI may not be directly related to the risk of VFs. Similar to the previous study conducted by Luo et al. (5) in women from the UK, we found that OB women and women with AO had a similar risk of VFs compared with NW women. We should not forget that women with VFs tend to have a higher level of height loss, which may overestimate the BMI level. Conversely, BMI does not reflect the changes in body composition in older people, including the loss of muscle and increase in fat, even if the person does have too much body fat. Therefore, BMI might not be an appropriate parameter to evaluate the relationship between obesity and VF risk, and further studies are needed to investigate the influence of other measurements of obesity on VF risk. Some studies have revealed that body composition helps to determine the difference in bone status between VF and non-VF participants (30, 31). Kuo et al. (31) found the prevalence of VFs is higher in postmenopausal women with increased BMI and fat percentage and in those with loss of lean mass. It’s reported that the risk of having osteoporosis was double in individuals with sarcopenia than in normal individuals (32). Together with the significantly positive associations between ALM and bone parameters in our study, these results emphasized the importance of body composition in the evaluation of VF risk. Unfortunately, there is a lack of sufficient data about body composition in our present study, except for ALM, to further investigate the relationship between obesity and VFs. It is commonly agreed that bone quality and bone strength are not directly related to the risk of fractures, which are also influenced by several other factors. Obesity is associated with several endocrine changes that could directly or indirectly affect bone metabolism. In addition to the lower bone turnover rate (10), obesity has been associated with increased levels of leptin and decreased IGF-1 (33, 34), which have a complex relationship with BMD and bone metabolism. The increased levels of pro-inflammatory cytokines in OB individuals are inversely associated with BMD and positively associated with bone resorption (35). Unfortunately, biochemical measurements of inflammatory cytokines, IGF-1, and leptin were not available in our study, and these may help to better clarify the relationship between obesity and bone health. Owing to the relatively small sample size, no significant difference in falling among groups was found in our study. Various studies have reported that the risk of falling in OB people is significantly increased (36, 37), due to postural instability (37), poorer balance capacity, and loss of muscle mass and strength (38). In addition, obesity is associated with various diseases, which can be associated with general weakness and peripheral neuropathy, predisposing individuals to falling (4).

There are several limitations in our present study. Given the cross-sectional design, we can determine that there is an association between obesity and bone microarchitecture but not a causal relationship. Second, morbid obesity may have an influence on the association between obesity and bone microarchitecture. The sample size of women with extremely elevated BMI is very small, making it difficult to investigate whether the favorable microarchitecture would reach a plateau or even be reversed in morbidly obese individuals. Third, we did not measure the body composition or fat distribution, which may provide more information about the relationship between obesity and bone health. Further analyses of the relationship between body composition and HR-pQCT parameters are needed. Finally, we did not perform microstructural finite-element analysis (μFEA) to estimate bone stiffness and strength in this study. Future studies are needed to fill this research gap.

## Conclusion

We have observed significant improvements in bone geometry and bone microarchitecture at the radius and tibia in obese postmenopausal Chinese women, synchronously with the increased aBMD at several skeletal sites. Except for a lower level of osteocalcin in the OB group, no significant differences in BTMs were found among the groups. Compared with body weight, ALM may explain greater variance in the improvement of bone microarchitecture parameters.

## Data Availability Statement

The original contributions presented in the study are included in the article/**Supplementary Material**. Further inquiries can be directed to the corresponding author.

## Ethics Statement

The studies involving human participants were reviewed and approved by The ethics committee of Peking Union Medical College Hospital. The patients/participants provided their written informed consent to participate in this study.

## Author Contributions

WX designed the study and revised the manuscript. WQ analyzed all data and drafted the manuscript. WQ, YJ, WL, YC, RJ, QP, OW, ML, XX, WY, and WX collected all the data used in this study. WQ and WX are responsible for the integrity of the data analysis. All authors read and approved the final manuscript.

## Funding

This study was funded by the National Natural Science Foundation of China (No.81900811, No.81170805, and No.81670714), and the Chinese Academy of Medical Sciences-CAMS Innovation Fund for Medical Sciences (CAMS-2016–12 M-3–003). Part of the study was supported by Merck Sharp & Dohme China, Hangzhou, China.

## Conflict of Interest

The authors declare that the research was conducted in the absence of any commercial or financial relationships that could be construed as a potential conflict of interest.

## Publisher’s Note

All claims expressed in this article are solely those of the authors and do not necessarily represent those of their affiliated organizations, or those of the publisher, the editors and the reviewers. Any product that may be evaluated in this article, or claim that may be made by its manufacturer, is not guaranteed or endorsed by the publisher.
