# Supplementary material for: Bone Microarchitecture in Obese Postmenopausal Chinese Women: The Chinese Vertebral Osteoporosis Study (ChiVOS)
Source: Front Endocrinol (Lausanne). 2022 Jul 5;13:891413. doi: 10.3389/fendo.2022.891413 (PMC9294215; doi:10.3389/fendo.2022.891413)
Supplement: Supplementary file 1 [file Table_1.docx]

**Supplemental Table 1**. Clinical characteristics in abdominal obesity (AO) or non-abdominal obesity (Non-AO) women.

|  | **AO (n=166)** | **Non-AO (n=77)** | ***p*-value** |
| --- | --- | --- | --- |
| **Age (years)** | 68.0(64.0-74.0) | 65.0(59.0-74.0) | 0.16 |
| **Height (cm)** | 155.04±6.58 | 155.09±6.13 | 0.962 |
| **Weight (kg)** | 93.8(88.95-99.85) | 55.20±6.89 | **<0.001** |
| **Waistline (cm)** | 66.0(60.0-74.0) | 78.8(75.0-83.25) | **<0.001** |
| **BMI (kg/m^2^)** | 27.3(25.5-29.8) | 22.8(21.1-24.5) | **<0.001** |
| **Age of menopause (years)** | 50.0(47.0-53.0) | 50.08±3.79 | 0.559 |
| **Falls (happened after age 50), n (%)** | 73/166(43.98%) | 29/77(37.66%) | 0.316 |
| **Falls (≥2 in the last year), n (%)** | 7/166(4.22%) | 6/77(7.79%) | 0.398 |
| **Any kinds of fractures (≥2 happened after age 50), n (%)** | 7/166(4.22%) | 3/77(3.90%) | >0.99 |
| **Vertebral fractures, n (%)** | 34/166(20.48%) | 10/77(12.99%) | 0.158 |
| **Long time use of glucocorticoid (≥3 months), n (%)** | 9/166(5.42%) | 2/77(2.60%) | 0.51 |
| **Long time use of HRT (≥3 months), n (%)** | 9/166(5.42%) | 4/77(5.19%) | >0.99 |
| **Anti-osteoporosis drugs (≥3 months), n (%)** | 5/166(3.01%) | 2/77(2.60%) | >0.99 |
| **ALM (kg)** | 15.60(14.44-17.21) | 14.35(12.95-15.19) | **<0.001** |
| **SMI (kg/m)** | 6.49(6.14-6.99) | 5.89±0.61 | **<0.001** |
| **Sarcopenia, n (%)** | 4/166(2.41%) | 18/77(23.38%) | **<0.001** |
| **Maxima of grip strength (kg)** | 21.15(16.9-25.78) | 22.33±4.68 | 0.142 |

1. Data presented as mean±SD or median (interquartile range) or percentages.

2. Significant values are shown in bold.

3. Abbreviations: BMI= body mass index; HRT: hormone-replacement therapy; ALM= appendicular lean mass; SMI= skeletal muscle index (SMI = ALM (kg) / height (m)).

**Supplemental Table 2**. aBMD and biochemical characteristics in women with abdominal obesity (AO) or non-abdominal obesity (Non-AO).

|  | **AO (n=166)** | **Non-AO (n=77)** | ***p*-value** |
| --- | --- | --- | --- |
| **Lumbar spine aBMD (g/cm^2^)** | 1.077±0.18 | 0.997(0.875-1.098) | **0.001** |
| **Femur neck aBMD (g/cm^2^)** | 0.793±0.14 | 0.758±0.11 | 0.064 |
| **Total hip aBMD (g/cm^2^)** | 0.854±0.20 | 0.818±0.12 | **0.022** |
| **TBS (L1-L4)** | 1.30±0.09 | 1.32±0.08 | **0.027** |
| **Ca(mmol/L)** | 2.32±0.08 | 2.32±0.06 | 0.63 |
| **P(mmol/L)** | 1.18±0.14 | 1.25±0.11 | **<0.001** |
| **ALP(U/L)** | 83.0(71.0-100.0) | 79.0(69.0-86.5) | 0.13 |
| **Total 25OHD(ng/ml)** | 14.78(12.0-19.03) | 16.58(13.25-23.5) | **0.009** |
| **PTH(pg/ml)** | 39.21(31.56-48.34) | 34.99(28.47-44.87) | **0.02** |
| **β-CTX(ng/ml)** | 0.34(0.23-0.46) | 0.38(0.26-0.47) | 0.229 |
| **P1NP(ng/ml)** | 51.43(39.72-63.33) | 55.36(43.41-71.22) | 0.077 |
| **Osteocalcin(ng/ml)** | 15.03(11.53-19.58) | 17.81(14.26-21.51) | **0.003** |

1. Data presented as mean±SD or median (interquartile range).

2. Significant values are shown in bold.

3. Abbreviations: TBS=trabecular bone score; ALP= alkaline phosphatase; total 25OHD= total 25-hydroxyvitamin D; PTH=parathyroid hormone; β-CTX=beta-C-terminal telopeptide; P1NP=N-terminal propeptide of type I procollagen.

4. Normal reference ranges: Ca: 2.13-2.70mmol/L; P: 0.81-1.45mmol/L; ALP: 50–135U/L; total 25OHD: 30-50ng/ml; PTH: 15.0-65.0pg/ml; β-CTX: menopause to 70y: 0.10–0.79ng/ml, >70y: 0.11–0.86ng/ml; P1NP: for premenopausal women: 15.1-58.6ng/ml.

**Supplemental Table 3**. Microarchitecture characteristics of postmenopausal women with abdominal obesity (AO) and non-abdominal obesity (Non-AO).

| HR-pQCT | Radius | | | Tibia | | |
| --- | --- | --- | --- | --- | --- | --- |
|  | **AO(n=166)** | **Non-AO(n=77)** | ***p*-value** | **AO(n=166)** | **Non-AO(n=77)** | ***p*-value** |
| Geometric features |  |  |  |  |  |  |
| Tt.Ar(mm^2^) | 252.80±43.21 | 239.71±32.82 | **0.01** | 549.25(487.43-637.6) | 637.61±90.40 | 0.143 |
| Ct.Ar(mm^2^) | 51.96±10.57 | 49.63±9.60 | 0.102 | 566.96±105.20 | 491.96±18.56 | **0.002** |
| Tb.Ar(mm^2^) | 203.95±44.71 | 193.58±34.52 | **0.049** | 100.82±20.67 | 550.78±95.84 | 0.253 |
| Volumetric BMD |  |  |  |  |  |  |
| Tt.vBMD(mg HA/cm^3^) | 252.1(201.0-305.75) | 245.0(209.8-300.95) | 0.791 | 219.9(185.95-261.75) | 215.37±54.77 | 0.191 |
| Tb.vBMD(mg HA/cm^3^) | 90.5(74.35-124.4) | 92.95±36.30 | 0.31 | 118.4±35.62 | 110.92±36.19 | 0.131 |
| Ct.vBMD(mg HA/cm^3^) | 892.3(829.3-944.5) | 888.10±66.08 | 0.701 | 826.4±70.44 | 835.12±67.81 | 0.364 |
| Microarchitecture measurements |  |  |  |  |  |  |
| Tb.BV/TV | 0.14(0.12-0.18) | 0.13(0.11-0.17) | 0.333 | 0.19±0.04 | 0.18±0.05 | 0.095 |
| Tb.N(1/mm) | 1.12±0.31 | 1.06±0.28 | 0.143 | 1.11(0.99-1.26) | 1.09±0.22 | 0.265 |
| Tb.Th(mm) | 0.22(0.21-0.23) | 0.22(0.21-0.23) | 0.328 | 0.24(0.23-0.26) | 0.24(0.23-0.25) | **0.009** |
| Tb.Sp(mm) | 0.87(0.73-1.06) | 0.89(0.77-1.18) | 0.211 | 0.89(0.77-1.02) | 0.89(0.80-1.09) | 0.378 |
| Tb.1/N.SD(mm) | 0.35(0.29-0.50) | 0.37(0.29-0.58) | 0.344 | 0.37(0.32-0.45) | 0.37(0.32-0.48) | 0.72 |
| Ct.Th(mm) | 0.93(0.76-1.07) | 0.91±0.20 | 0.504 | 3.4(2.5-4.6) | 1.13±0.25 | **0.024** |
| Ct.Po(%) | 0.7(0.4-1.0) | 0.6(0.4-1.0) | 0.531 | 1.20(1.04-1.39) | 3.37±1.60 | 0.137 |

1. Significant values are shown in bold.

2. Abbreviations: Tt.Ar= total area; Ct.Ar= cortical area; Tb.Ar= trabecular area; Tt.vBMD= total volume bone mineral density; Tb.vBMD= trabecular volume bone mineral density; Ct.vBMD= cortical volume bone mineral density; Tb.BV/TV= trabecular bone volume fraction; Tb.N= trabecular number; Tb.Th= trabecular thickness; Tb.Sp= trabecular separation; Tb.1/N.SD= trabecular inhomogeneity of network; Ct.Th= cortical thickness; Ct.Po= cortical porosity.
